# The effect of positive airway pressure therapy on neurocognitive functions, depression and anxiety in obesity hypoventilation syndrome

**DOI:** 10.1186/s40248-016-0071-2

**Published:** 2016-10-11

**Authors:** Serap Argun Baris, Dilek Tuncel, Cigdem Ozerdem, Huseyin Kutlu, Tugba Onyilmaz, Ilknur Basyigit, Hasim Boyaci, Fusun Yildiz

**Affiliations:** 1 Department of Pulmonary Diseases, Kocaeli University School of Medicine, Umuttepe, İzmit, Kocaeli Turkey; 2 Department of Neurology, Derince Training and Research Hospital, İzmit, Kocaeli Turkey; 3 Department of Pyschiatry, Kocaeli University School of Medicine, İzmit, Kocaeli Turkey; 4 Department of Pulmonary Diseases, Private Konak Hospital, İzmit, Kocaeli Turkey

**Keywords:** Obesity Hypoventilation Syndrome, Neurocognitive evaluation, Depression, Anxiety, SF-36, Positive airway pressure (PAP)

## Abstract

**Background:**

The aim of this study is to evaluate the presence of neurocognitive dysfunctions, depression and anxiety and the effect of positive airway pressure (PAP) therapy on these alterations in Obesity Hypoventilation Syndrome (OHS) patients.

**Methods:**

Ten healthy normal and obese controls, 10 OHS and 10 OSAS patients were included in the study. Short form-36, Beck Depression Scale and State-Trade Anxiety Inventory (STAI 1-2) were performed. Wisconsin Card Sorting Test (WCST), Montreal Cognitive Assessment Scale (MOCA), Enhanced Cued Recall (ECR) and Mini Mental Test (MMT) were used for neurocognitive evaluation. All tests were repeated after one night PAP therapy in OHS and OSAS groups.

**Results:**

OHS patients had the lowest scores of physical (PF) and social functioning (SF) in SF-36. The total number of persistent errors and incorrect answers were the highest in OHS group in WCST. The scores of MOCA, ECR and MMT were lower; depression and anxiety scores were higher in OHS group than in controls (*p* = 0,00). There was a significant increase in the completed categories in OHS after PAP therapy (*p* = 0,03). There were also significant increases in MOCA, ECR and MMT scores and significant decreases in depression and anxiety scores with respect to PAP therapy.

**Conclusions:**

Cognitive dysfunction, depression and anxiety are important under-recognized comorbidities in OHS. It is suggested that short term PAP therapy had positive effects on neurocognitive functions, depression and anxiety but further multicentre, prospective studies with large number of cases are needed to evaluate the effect of long term PAP therapy on these parameters.

## Background

Obesity is a global health problem with increasing prevalence. It is related to severe economic burden on health care services due to high mortality and morbidity [1]. Obesity Hypoventilation Syndrome is defined as a combination of obesity (BMI ≥ 30 kg/m^2^) and daytime hypercapnia in arterial blood gas analysis (PaCO_2_ > 45 mmHg) without other pathologies that cause hypoventilation [2]. The pathophysiology of OHS includes increased mechanic load on respiratory system, blunting of ventilatory drive, and inadequate chemoreceptor response to hypercarbia and hypoxemia. The hypercapnia and hypoxia induce pathologic effects that further worsen sleep-related breathing, resulting in a slowly progressive worsening of disease.

Comorbid diseases, especially the cardiac ones, are frequent in OHS. Neurocognitive dysfunction and psychiatric disorders such as depression and anxiety are some of probable comorbid diseases. Neurocognitive impairment is associated with limited creativity, work performance, quality of life, self esteem, and psychosocial functioning [3]. Psychiatric symptoms and cognitive dysfunction often occur in patients with chronic respiratory diseases such as asthma and chronic obstructive pulmonary disease (COPD) [4]. Cognitive dyfunctions, depression and anxiety are also frequent in Obstructive Sleep Apnea Syndrome (OSAS) patients [5–8]. OHS and OSAS have similar clinical characteristics including obesity, sleepiness and snoring. While there are many studies evaluating the role of OSAS or obesity on neurocognitive functions and psychiatric disorders, there are no studies evaluating these parameters in OHS patients. As a major component of OHS, obesity is related to behavioral problems, depression and anxiety [9–11]. Morbidly obese patients are described as depressed, anxious and with impaired quality of life. These people are exposed to physical, psychological and social consequences of obesity and they have high risk for psychiatric disorders [12].

Positive airway pressure therapy is first line therapy in both OHS and OSAS. While there are many studies evaluating the role of PAP therapy on neurocognitive functions and psychiatric disorders in OSAS patients [13–16], there are no studies evaluating the role of PAP therapy on neurocognitive functions in OHS patients. The aim of this study is to evaluate the quality of life, presence of neurocognitive dysfunctions, depression and anxiety and the effect of positive airway pressure (PAP) therapy on these parameters in OHS patients.

## Methods

### Study population

Ten healthy normal and obese controls, 10 OHS and 10 OSAS patients were included in the study between October 2014 and December 2015. It was a hospital based study.

#### Inclusion criteria

Individuals who were advised to use PAP therapy for the diagnoses of OHS and OSAS were included in the study. All patients were evaluated by PSG and arterial blood gas analysis (ABG). Isolated OHS and OSAS patients were included, while overlap cases with both hypercapnia and obstructive sleep apnea findings at PSG were excluded.

Healthy normal and obese controls with snoring and increased Epworth scale also underwent PSG and ABG analysis for excluding OSAS and OHS. All participants were informed about the study aim and gave written informed consent.

#### Exclusion criteria

Individuals with acute decompansated OHS, age  < 18 years, barriers to PAP therapy due to facial deformities, neurologic and psychiatric disorders and hypercapnic diseases such as chronic obstructive pulmonary disease, respiratory failure related to chest wall deformities and neuromuscular disease were excluded from the study.

### Study plan

There were ten healthy normal and ten obese controls, 10 OHS and 10 OSAS patients, totally forty participants in the study. Initially, demographic characteristics of the participants including gender, BMI, smoking history and Epworth Sleepiness Scale (ESS) results were recorded. Quality of life was assessed by Short form-36 (SF-36). Depression and anxiety questionnaires and neurocognitive assessment tests were performed in the morning by psychologists. Wisconsin Card Sorting Test (WCST), Montreal Cognitive Assessment Scale (MOCA), Enhanced Cued Recall (ECR) and Mini Mental Test (MMT) were used for neurocognitive evaluation.

Patients with OHS and OSAS underwent PAP therapy in the same day. Continuous positive airway pressure (CPAP) therapy was used for OSAS patients while bi-level continuous positive airway pressure (BIPAP) therapy was used for OHS patients. Mean efficient CPAP pressure was 6 or 7 cm H_2_0 in OSAS patients. For BIPAP therapy, inspiratory (IPAP) and expiratory (EPAP) pressures were 15 cmH_2_O and 5 cmH_2_O respectively. All tests were repeated after one night PAP therapy in these groups.

#### * Short form-36 (SF-36)

The SF-36 is widely used to measure health status and quality of life. It includes eight domains as follows: physical functioning (PF), role limitations due to physical problems (RP), bodily pain (BP), general health perception (GH), energy/vitality (VT), role limitations due to emotional problems (RE), social functioning (SF), and mental health (MH). Each domain is transformed onto a score from 0 (worst possible health) to 100 (best possible health).

#### * Montreal cognitive assessment scale-MOCA

MOCA assesses different cognitive dimensions including orientation, attention, concentration, executive functions, memory, language, and visual-spatial skills. Total score of MOCA < 26 points is considered to be cognitive dysfunction.

#### * Beck depression scale-BDS

The Beck Depression Inventory is a self-rating scale for measuring symptoms of depression. The lowest score is 0 and the highest score is 63. Twelve points and above is considered to be depression.

#### * State-trade anxiety inventory, STAI 1-2

*STAI 1* indicates how individuals feel at a given moment or condition. *STAI 2* determines how individuals feel regardless of their conditions. The lowest score is 20 and the highest one of the scale is 80 for both state and trade anxiety inventory. The increase in test scores correlated with severity of anxiety.

#### * Mini mental test (MMT)

Mini Mental Test is a tool that can be easily applied. MMT evaluates the 5 main functions including orientation, registration memory, attention and recall, calculation and language. The highest score of the test is 30. Total score of MMT ≥ 24 points is considered to be normal, while <24 points as cognitive dysfunction.

### Statistical analyses

All statistical analyses were performed using IBM SPSS for Windows version 20.0 (SPSS, Chicago, IL, USA). Kolmogorov-Smirnov Tests were used to test the normality of data distribution. Continuous variables were expressed as median (25.percentile-75.percentile), and categorical variables were expressed as counts (percentage). Comparisons of continuous paired variables were performed using the Wilcoxon *t* test and comparisons of continuous variables between groups were performed using the Kruskal Wallis Analysis of Variance and Dunn’s Post Hoc Test. Comparisons of categorical variables between the groups were performed using the Yates’s and Monte Carlo chisquare test. A two-sided *p* < 0.05 was considered statistically significant.

## Results

**Fig. 1:**
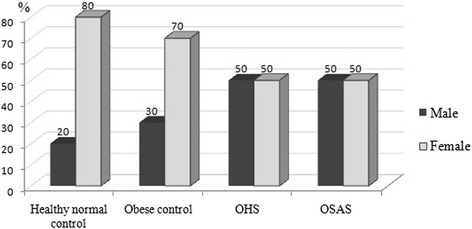
The distribution of gender according to groups (%)

There were 25 females (62.5 %) and 15 males (37.5 %), totally 40 individuals. The distrubition of the gender according to groups is shown in Fig. 1. The mean age was 45.7 ± 14.6 years and the mean BMI was 38.03 ± 10.3 kg/m^2^. The mean age was lower in control groups than in OHS and OSAS groups and the difference was significant (*p* = 0.003). However, the mean ages of OHS and OSAS groups were similar. BMI was similar in obese control, OHS and OSAS groups. A significant difference of Epworth score was found between healthy normal controls and patient groups (OHS and OSAS) (*p* = 0.007 and *p* = 0.002). There was also a significant difference of Epworth score between obese controls and OSAS group (*p* = 0.027). The median of smoking pack/years in OHS group was higher than in other groups (Table 1).

**Table 1:** Demographic characteristics of the groups

Group			Age	BMI	Smoking pack/years	Epworth score
Healthy normal control (*N* = 10)	Median		31.5^a^	25.6^a,b,c^	0,5^a^	1^a,b^
	Percentile	25	26	22.4	0	0
		75	51.25	27.2	7	4
Obese control (*N* = 10)	Median		38^b^	39.6^a^	4^b^	1.5
	Percentile	25	27.25	37.04	0	1
		75	47.75	48.05	13.5	6
OHS (*N* = 10)	Median		56.5^a, b^	44.97^b^	18^a,b,c^	10^a^
	Percentile	25	51.75	40,2	0	3.75
		75	64	54.7	67.5	15.25
OSAS (*N* = 10)	Median		52	37.6^c^	3.5^c^	9.5^b^
	Percentile	25	35.75	32.7	0	5.5
		75	59.5	41.96	26.25	18.5

Comparison of groups: Statistically significant differences (*p* < 0.05) were summarized. Age: a. Healthy normal control and OHS, *p* = 0.015 b. Obese control and OHS, *p* = 0.013

BMI: a. Healthy normal control and obese control, *p* = 0.04 b. Healthy normal control and OHS, *p* = 0.001 c. Healthy normal control and OSAS, *p* = 0.001

Smoking package: a. Healthy normal control and OHS, *p* = 0.001 b. Obese control and OHS, *p* = 0.023 c. OHS and OSAS, *p* = 0.032

Epworth score: a. Healthy normal control and OHS, *p* = 0.007 b. Healthy normal control and OSAS, *p* = 0.002 c. Obese control and OSAS, *p* = 0.027

There were significant differences in SF-36 subscales including physical functioning (PF) (*p* = 0.03), general perception of health (GH) (*p* = 0,03) and social functioning (SF) (*p* = 0,04) between the groups. OHS patients had the lowest scores of PF and SF (Table 2).

**Table 2:** The results of SF-36 according to groups

0	Healthy normal control		Obese control		OHS		OSAS		P
	Med.	Percentile 25/75	Med.	Percentile 25/75	Med.	Percentile 25/75	Med.	Percentile 25/75	
PF	95^a^	76.25 100	77.5	26.25 91.25	40^a^	28.75 76.25	55	40 73,75	0.03
RP	100	25 100	50	0 100	12.5	0 62.5	37.5	0 100	0.13
BP	100	56.75 100	72	22 100	62,5	38.5 100	90	36,25 100	0.66
GH	76^a^	56.5 91.75	32.5^a^	18.75 78.25	57.5	30 74.5	41	28,75 53,25	0.03
VT	65	43.75 82.5	57.5	38.75 75	60	18.75 81.25	45	28,75 66,25	0.46
SF	87,5^a^	71.88 100	62.5	25 87.5	37.5^a^	12.5 78.13	56.25	21,88 81,25	0.04
RE	100	33.3 100	33.3	0 100	16.5	0 49.98	33.3	0 100	0.09
MH	68	55 80	64	52 77	58	42 77	56	42 60	0.28

Comparison of groups: Statistically significant differences (*p* < 0.05) were summarized

PF: a. Healthy normal control and OHS, *p* = 0.03

SF: a. Healthy normal control and OHS, *p* = 0.04

GH: a. Healthy normal control and obese control, *p* = 0.03

PF: Physical functioning; RP: Role limitation due to physical problems; BP: Bodily pain; GH: General perception of health; VT: Energy and vitality; SF: Social functioning; RE: Role limitation due to emotional problems; MH: Mental health

The number of completed categories and correct answers was the lowest in OHS group in WCST. Furthermore, the total number of persistent errors and incorrect answers was the highest in OHS group. There were significant differences in the total number of completed categories, correct numbers, persistent errors and percentage of conceptual level responses among the groups. The numbers of completed categories were significantly lower in OHS and OSAS than in healthy normal controls (*p* = 0.01; *p* = 0.03) (Table 3).

**Table 3:** Winconsin Cart Sorting Test results of the groups

	Healthy normal control		Obese control		OHS		OSAS		P
	Med.	Perc. 25/75	Med.	Perc. 25/75	Med.	Perc. 25/75	Med.	Perc. 25/75	
Trials administered	103.5	81 128	117	107 28	128	128 128	128	102.5 128	0.04
Total number of correct answers	76	63 88.25	78	62.5 84.5	56	43.5 78.5	66	52.5 72.5	0.12
Total number of incorrect answers	23.5	12.75 37.25	32	24.5 64	72	49.5 84.5	62	28 75.5	0.01
Total number of persistent errors	13,5	4.75 19.25	16	9 28.5	53	18.5 65.5	24	9.5 40	0.03
Number of completed categories	6^a,b^	5.5 6	6	2 6	1^a^	1 3	2^b^	1 4.5	0.01
Percentage of persistent errors	9.5	7.75 16	12	11 23	14	12.5 20	20	12 29	0.15
The percentage of conceptual level responses	73	65.25 80.75	64	35.5 71.5	27	18 50.5	28	21.5 68.5	0.01
Learning to learn scores	0.26	-1.62 2.12	1.97	-2.06 2	-10.5	1 1.5	-6.85	-28.3 0.2	0.24

Comparison of groups: Statistically significant differences (*p* < 0.05) were summarized

The number of completed categories: a. Healthy normal control and OHS, *p* = 0.01 b. Healthy normal control and OSAS, *p* = 0.03

**Fig. 2:**
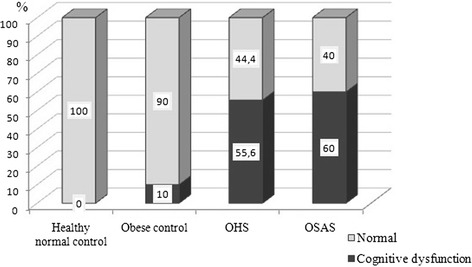
Comparison of cognitive dysfunction according to MMT scores among the groups (%)

The scores of MOCA and ECR tests were the lowest in OHS group. MMT scores were lower in both OHS and OSAS groups than in controls and the difference was statistically significant (*p* = 0.00) (Table 4). Nearly half of the OHS patients had cognitive dysfunction according to cut off value (MMT < 24 points) (Fig. 2).

**Table 4:** Comparison of neurocognitive assessment test scores among the groups

	Healthy normal control		Obese control		OHS		OSAS		
	Med.	Perc. 25/75	Med.	Perc. 25/75	Med.	Perc. 25/75	Med.	Perc. 25/75	P
MOCA	29.5^a,c^	22.75 30	28^b,d^	27 29.5	18^a,b^	16 19.5	20.5^c,d^	17 25	0.00
ECR	48^a,c^	48 48	48^b,d^	45 48	35^a,b^	31 38.5	36^c,d^	29.75 37.25	0.00
MMT	29^a,b^	27.5 30	28^b,c^	26.25 30	22^a^	19.5 26.5	22^b,c^	19.75 26.25	0.00

Comparison of groups: Statistically significant differences (*p* < 0.05) were summarized

MOCA: a. Healthy normal control and OHS, *p* = 0.003 b. Obese control and OHS, *p* = 0.003 c. Healthy normal control and OSAS, *p* = 0.047 d. Obese control and OSAS, *p* = 0.043

ECR: a. Healthy normal control and OHS, *p* = 0.002 b. Obese control and OHS, *p* = 0.007 c. Healthy normal control and OSAS, *p* = 0.001 d. Obese control and OSAS, *p* = 0.004

MMT: a. Healthy normal control and OHS, *p* = 0.008 b. Healthy normal control and OSAS, *p* = 0.003 c. Obese control and OSAS, *p* = 0.035

There was a significant difference in MMT score in subgroup analysis (*p* = 0.00). While there was no significant difference in MMT score in OHS and OSAS groups, it was found that the difference was statistically significant between healthy normal control and OHS group (*p* = 0.008). Also, subgroup analyses of MOCA and ECR showed significant differences between OHS and control groups (*MOCA:* OHS-Healthy normal control *p* = 0.003; OHS- Obese control *p* = 0.003; *ECR:* OHS-Healthy normal control *p* = 0.002; OHS-Obese control *p* = 0.007). There were significant differences in MOCA and ECR scores between OSAS and control groups (*MOCA*: OSAS- Healthy normal control *p* = 0.047; OSAS-Obese control *p* = 0.043; *ECR*: OSAS- Healthy normal control *p* = 0.001; OSAS-Obese control *p* = 0.004).

**Fig. 3:**
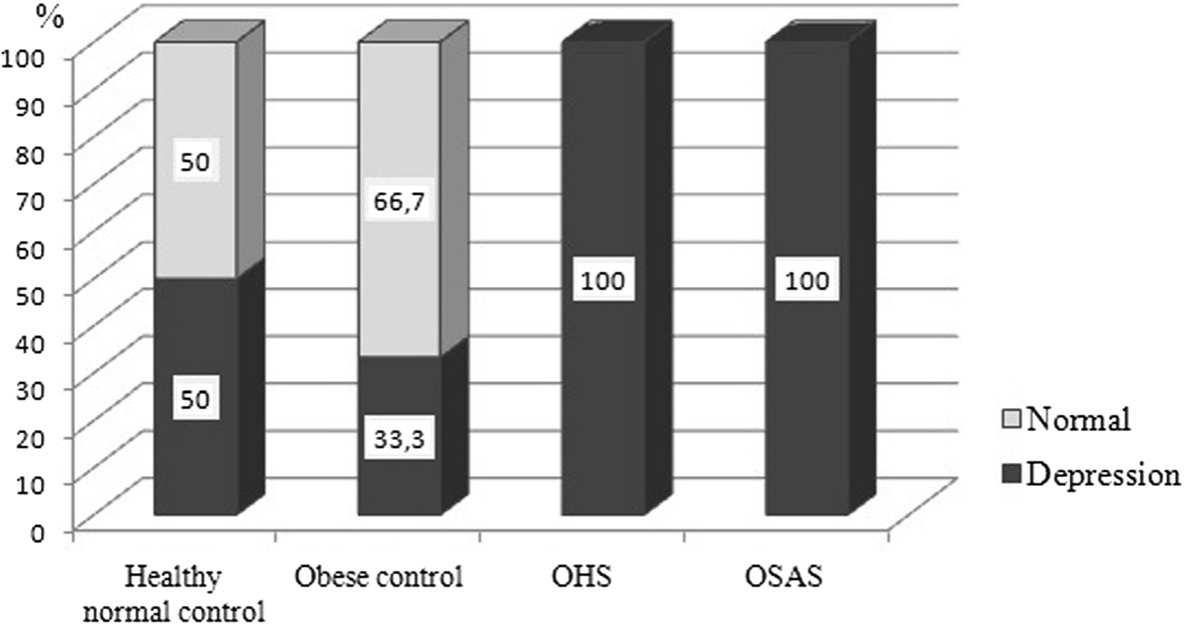
The distribution of depression among the groups (%)

**Fig. 4:**
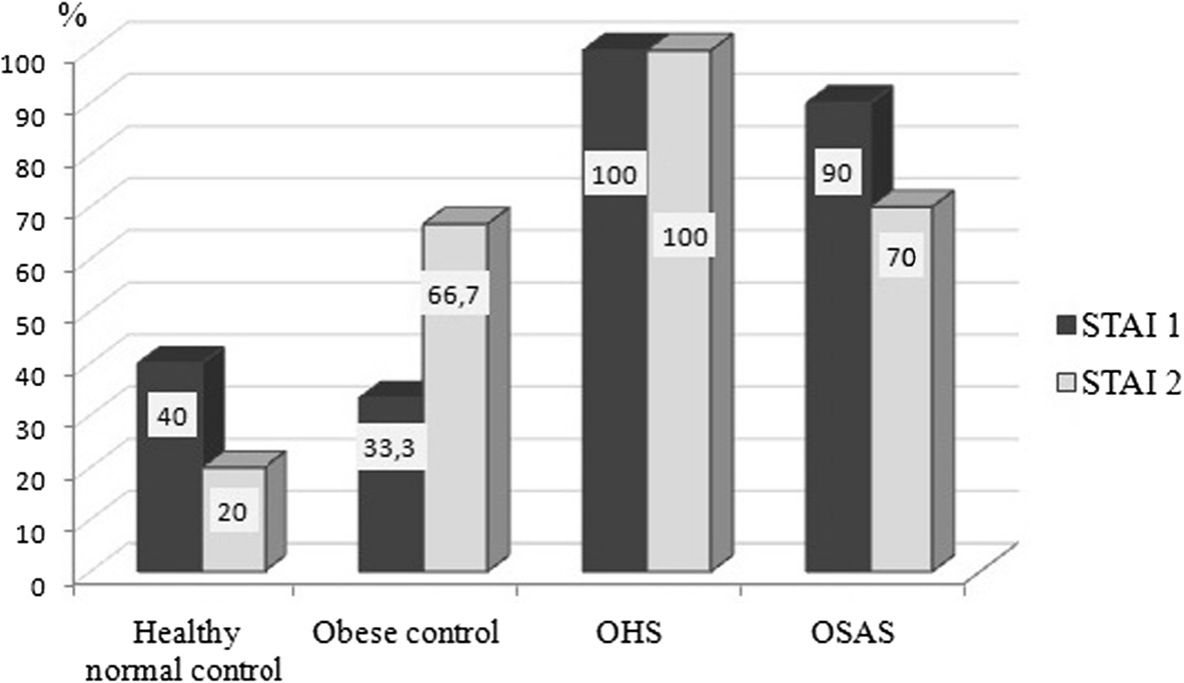
Presence of state and trade anxiety among the groups (%)

Depression and anxiety scores were higher in OHS group than in controls (*p* = 0.00) (Table 5). The cut off values for depression and anxiety were respectively 12 points and 44 points. All of the participants had high depression scores in both OHS and OSAS groups (Fig. 3). Furthermore, the percentage of state and trade anxiety was the highest in OHS group (Fig. 4). While there was no significant difference in Beck Depression score in OHS and OSAS groups at subgroup analysis, it was found that the difference was statistically significant between OHS and control groups (OHS- Healthy normal control, *p* = 0.014; OHS-Obese control, *p* = 0.005). Subgroup analysis of STAI-1 showed significant differences between OHS and control groups (OHS-Healthy normal control *p* = 0.025; OHS- Obese control *p* = 0.007). In addition, there were significant differences in STAI-2 scores between OHS and obese control group (*p* = 0.033) and OSAS and obese controls (*p* = 0.04).

**Table 5:** Comparison of depression and anxiety scores (state and idem) of the groups

	Healthy normal control		Obese control		OHS		OSAS		
	Med.	Perc. 25/75	Med.	Perc. 25/75	Med.	Perc. 25/75	Med.	Perc. 25/75	p
Beck depression	12^a^	5.5 16.5	7^b,c^	4.5 19.5	26^a,b^	21.5 30	25^c^	17 33	0.00
STAI 1	42^a,c^	33.25 50.25	37^b,d^	34 45.5	55^a,b^	50 65.5	58^c,d^	48.75 62.25	0.00
STAI 2	38.5	36 43.5	44^a,b^	35.5 52	51^a^	47.5 55	54^b^	43 58	0.01

Comparison of groups: Statistically significant differences (*p* < 0.05) were summarized

BDS: a. Healthy normal control and OHS, *p* = 0.014 b. Obese control and OHS, *p* = 0.005 c. Obese control and OSAS, *p* = 0.023

STAI-1: a. Healthy normal control and OHS, *p* = 0.025 b. Obese control and OHS, *p* = 0.007 c. Healthy normal control and OSAS, *p* = 0.024 d. Obese control and OSAS, *p* = 0.007

STAI-2: a. Obese control and OHS, *p* = 0.033 b. Obese control and OSAS, *p* = 0.04

The number of completed categories in WCST was increased after one night PAP in both OHS and OSAS groups (*p* = 0.03) (Table 6). In addition,  there were significant increases in MOCA (*p* = 0.01), ECR (*p* = 0.01) and MMT (*p* = 0.04) scores and significant decreases in depression (*p* = 0.01) and anxiety scores (STAI 1 *p* = 0.05; STAI 2 *p* = 0.02) with respect to PAP therapy (Tables 7 and 8).

**Table 6:** The effect of PAP therapy on WCST scores in OHS and OSAS patients

	OHS					OSAS				
	Before PAP therapy		After PAP therapy			Before PAP therapy		After PAP therapy		
	Med.	Pers. 25/75	Med.	Pers. 25/75	p	Med.	Pers. 25/75	Med.	Pers. 25/75	P
Trials administered	128	128 128	128	128 128	1.0	128	102.5 128	128	128 128	0.18
Total number of correct answers	56	43.5 78.5	83	53 94.75	0.12	66	52.5 72.5	71	67 84.5	0.09
Total number of incorrect answers	72	49.5 84.5	45	33.25 75	0.12	62	28 75.5	57	37.5 61	0.58
Total number of persistent errors	53	18.5 65.5	26	17.5 36	0.24	24	9.5 40	25	11 29.5	0.9
The number of completed categories	1	1 3	3	2.25 5	0.03	2	1 4,5	4	2.5 6	0.05
The percentage of conceptual level responses	27	18 50.5	53	23.75 66	0.12	28	21.5 68.5	50	40 75.5	0.17
Learning to learn scores	-10.5	1 1.5	-9.5	-21.7 0.54	1	-6.85	-28.3 0.2	-3.7	-12.6 1.49	0.07

**Table 7:** The effect of PAP therapy on MOCA, ECR and MMT scores in OHS and OSAS patients

	OHS					OSAS				
	Before PAP therapy		After PAP therapy			Before PAP therapy		After PAP therapy		
	Med.	Pers. 25/75	Med.	Pers. 25/75	p	Med.	Pers. 25/75	Med.	Pers. 25/75	p
MOCA	18	16 19.5	27.5	24 30	0.01	20.5	17 25	30	24.5 30	0.01
ECR	35	31 38.5	48	48 48	0.01	36	29.75 37.25	48	47 48	0.01
MMT	22	19.5 26.5	26.5	24.25 29.75	0.04	22	19.75 26.25	28.5	24.75 29.25	0.005

**Table 8:** The effect of PAP therapy on depression and anxiety scores in OHS and OSAS patients

	OHS					OSAS				
	Before PAP therapy		After PAP therapy			Before PAP therapy		After PAP therapy		
	Med.	Pers. 25/75	Med.	Pers. 25/75	p	Med.	Pers. 25/75	Med.	Pers. 25/75	p
Beck depression	26	21.5 30	14	6.75 17.75	0.01	25	17 33	11	4 18.5	0.02
STAI 1	55	50 65.5	48	43.5 51	0.05	58	48.75 62.25	42	38.5 49.5	0.04
STAI 2	51	47.5 55	38.5	36.25 41.25	0.02	54	43 58	36	34.9 39.5	0.02

## Discussion

This study demonstrated that OHS patients had the lowest scores of physical (PF) and social functioning (SF) in SF-36. The total number of persistent errors and incorrect answers was the highest in OHS group in WCST. The scores of MOCA, ECR and MMT were lower and depression/anxiety scores were higher in OHS patients than controls. There was a significant increase in completed categories in WCST after PAP therapy. Furthermore, there were significant increases in MOCA, ECR and MMT scores and significant decreases in depression and anxiety scores with respect to PAP therapy.

The Obesity Hypoventilation Syndrome is a chronic systemic disease characterized by comorbidities resulting in high mortality and morbidity rates. Respiratory, metabolic, hormonal and cardiovascular comorbidities are related with decreased daily activities and quality of life [17].

The prevalence of OHS in general population is unclear and data about OHS prevalence are mostly described in OSAS patients. It is estimated that prevalence ranges from 10 % to 38 % [18–20]. In a retrospective study, coexistence of OSAS and OHS were identified in 11% of patients [21]. Hypoventilation was found in 31 % of the obese subjects who did not have other reasons for hypercapnia [22]. It is suggested that OHS is expected to increase in near future depending on increasing obesity prevalence worldwide. In this study, OHS and OSAS patients were evaluated by polysomnography and arterial blood gas analysis. Isolated OHS and OSAS patients were enrolled while overlap patients were excluded from the study.

The Epworth Scale is a validated queastionnaire assessing the sleepiness of the patient. In Hida et al.’s study, the Epworth score was found to be worse in untreated OHS and OSAS patients than in matched healthy subjects [23]. Also Epworth scores of OHS patients were worse than in age and BMI matched OSAS group [21]. Similar to literature, the Epworth score was the highest in OHS group and the difference was significant (*p* = 0,007) in our study. On the other hand, there were no significant differences in age, BMI and Epworth scores between OHS and OSAS groups.

The median of smoking pack/years in OHS group was higher than in other groups. The half of the participants in OHS and OSAS were male while this ratio was 20 % for healthy normal controls and 30 % for obese controls. Smoking is more prevalent in men than in women in our country. As result, the difference might be explained by the age and gender distribution of the groups.

The hypercapnia and hypoxia induce pathologic effects that further worsen sleep-related breathing, resulting in a slowly progressive worsening of OHS. Health care costs are high and comorbidities are common in untreated patients. Psychiatric disorders and neurocognitive dysfunction are probable adversely affected conditions. Neurocognitive impairment restricts creativity, work performance, quality of life, self esteem, and psychosocial functioning [3]. Excessive sleepiness and altered circadian rhythms may negatively affect ability to learn, employment, interpersonal relations, and quality of life [24].

The SF-36 is widely used to measure health status and quality of life. The test is not specific to any illness or age group. The effects of sleep disorders on the quality of life have been documented in the literature [24]. However, the studies assessing quality of life in OHS patients are limited [21–23]. Untreated OHS and OSAS patients had worse results compared to matched healthy subjects on SF-36 subscales such as physical functioning, role limitations due to physical problems, general health perception, energy/vitality, role limitations due to emotional problems and social functioning [21]. SF-36 subscales such as physical functioning, role limitations due to physical problems, body pain, social functioning and role limitations due to emotional problems were the worst in OHS group in our study. The difference in physical and social functioning scores between OHS and healthy normal control was significant. Although these findings are consistent with the literature, the difference between healthy normal controls and OHS group may be affected by the lower mean age of control group. Therefore, it is suggested that prospective studies with large number of age matched cases are needed to support this findings.

Psychiatric comorbidities have an important role on quality of life [4]. Many authors draw attention to relationship between mental health and disease progression and treatment efficacy [25, 26]. It is reported that severity of the comorbidities is frequent in the coexistence of depression [27]. The existence of psychiatric symptoms in patients with respiratory diseases is higher compared to the controls in several studies [4, 25, 28, 29]. Depression and anxiety are common also in OSAS patients [5].

Obesity as a major component of the OHS has been investigated in many studies [9, 10]. It is reported that obesity is associated with behavioral problems, depression and anxiety. The symptoms of depression and anxiety are high in obese patients before the bariatric surgery [10]. Obese adolescents suffer from body dissatisfaction, anhedonia, low self-esteem, and depressive symptoms more often than normal-weight persons [11]. Morbidly obese patients are described as depressed, anxious, having poor impulse control, low self-esteem, and impaired quality of life. These people are exposed to physical, psychological and social consequences of obesity and they have high risk for psychiatric disorders [12].

To our knowledge, there are no studies evaluating depression and anxiety in OHS patients. All patients in OHS and OSAS groups had depressive symptoms in our study. Increased scores of Beck Depression Scale are correlated with the severity of depression. Median depression scores of healthy control, obese control, OHS and OSAS patients were 12 points, 7 points, 26 points and 25 points respectively in this study. However, as an interesting result, half of the healthy normal controls had depression according to cut off value since the median score of the healthy controls was equal to that of depression. It is suggested that high percentage of depression in healthy controls might be explained by female predominance and overdiagnosis of depression due to using a cut-off value instead of real scores.

State and trade anxiety scores were also highest in OHS group. While the depression and anxiety scores of OHS and OSAS groups were similar, there were significant differences in these parameters between OHS group and both normal and obese controls. Although there is no difference in BMI between obese controls and OHS patients, increased depression and anxiety scores of OHS patients suggest the role of various factors in addition to obesity. It is thought that OHS patients are required to assess the development of psychiatric disorders.

As in psychiatric disorders, cognitive function can be affected by chronic diseases. Although the prevalence of cognitive dysfunction in patients with OSAS is not fully known, cognitive dysfunctions including attention and memory impairment have been reported in the literature [6–8]. Various mechanisms have been identified in development of cognitive dysfunction in OSAS [6, 7]. The first of these mechanisms is excessive daytime sleepiness. Sleepiness plays a role in the development of cognitive dysfunction, especially in attention and executive functions [30, 31]. A second possible mechanism is intermittant hypoxemia [32]. Hypoxemia is found to be associated with reduced attention and processing speed [14, 33]. It is reported that cognitive functions are worse in hypoxemic OSAS patients than in non-hypoxemic ones [34]. Also the individual factors such as age and obesity have an important effect on cognitive dysfunction [35–37].

It is suggested that intermittant hypoxemia, daytime sleepiness and obesity may have an effect on neurocognitive dysfunction in OHS patients. While there are many studies evaluating the role of OSAS and obesity on neurocognitive functions, there is no study evaluating this relationship in OHS patients. In the present study, neurocognitive functions have been assessed by using various tests such as WCST, MMT, MOCA and ECR.

MMT is one of the most frequently used tests. It is reported that MMT was lower in OSAS than in controls in Kanbay et al.’s study [38]. In baseline assessment of this study, MMT scores were found to be lower in both OHS and OSAS groups than in controls and the difference was statistically significant (*p* = 0.00). Nearly fifty five percent of the OHS patients had cognitive dysfunction according to cut-off value (MMT < 24 points). As similar to MMT, MOCA and ECR were also significantly lower in OHS and OSAS patients with respect to controls. However, the results of MOCA, ECR and MMT were similar in both patient groups. The numbers of completed categories and correct answers were the lowest in OHS group in WCST. Also the total number of persistent errors and incorrect answers were the highest in OHS group. Perseveration means to insist on the same behavior even though it is said that it was wrong. Despite our small number of cases, the similar results of different neurocognitive tests supporting each others are remarkable. Individuals with cognitive impairment have a tendecy to misunderstanding the recommendations of the doctor [4]. Therefore, a correct and effective treatment of OHS should start with a multidisciplinary approach and all patients with OHS should be assessed for neurologic and psychiatric disorders.

Positive airway pressure therapy is the first line therapy in both OHS and OSAS [39, 40]. The role of PAP therapy on neurocognitive functions in OSAS patients has been evaluated in literature [7, 13], but there is no study assessing this relation in OHS patients. The results of these studies are controversial due to different sampling and methodology. While there was no significant difference in cognitive functions before and after PAP therapy [7, 41], some of these studies reported an improvement in attention, executive functions and short memory after CPAP therapy [14–16].

One of the main reasons for the different outcomes is the variable duration of PAP therapy (min: 1 week and max: 12 months). Bardwell et al. reported that there was a cognitive improvement after one week CPAP therapy [14]. There was an improvement in attention and speed of motor skills after CPAP therapy but the results were not different after 15 days or 4 months [16]. A positive relationship between the duration of PAP therapy and continuous improvement in attention was only shown in Munoz et al.'s study [15]. The shortest duration of PAP therapy in literature was one night. Our clinical observations and experiences showed that OHS patients were unhappy, sleepy, hopeless and reluctant to therapy. They had limited verbal communication due to sleepiness during the hospitalization. It was observed that they had a positive attitude about the management after even one night PAP therapy. Furthermore, they were hopeful and willing to live since they had a good sleep. This experience was the cornerstone of our study. So we aimed to evaluate the acute effects of PAP therapy on neurocognitive functions.

There were significant increases in MOCA, ECR MMT, and WCST scores after one night PAP therapy. However, only one item (the number of completed categories) of the WCST had a significant improvement after treatment. It might be explained by the relatively small size of our study population and short duration of PAP therapy. In addition, there were significant decreases in depression and anxiety scores with respect to PAP therapy. These findings suggest that PAP therapy has positive effects on neurocognitive functions and psychiatric symptoms even in a short period. However, these are chronic disorders and we think that positive results after one night PAP therapy do not fully reflect the improvement. It is suggested that further prospective studies with large number of cases are needed to evaluate the effect of long term PAP therapy on these parameters. It is planned to control neurocognitive assessment in the sixth month of the PAP therapy in OHS and OSAS groups for follow up.

To our knowledge, this is the first study evaluating the neurocognitive functions, depression/anxiety and the role of PAP therapy on these parameters in OHS patients. Beside this, the limited size of the patient groups, the presence of younger control group and the assessment of short term effects of PAP therapy are the limitations of this study.

## Conclusions

Cognitive dysfunction, decreased quality of life, depression, and anxiety are important recognized comorbidities in OHS. The scores of MOCA, ECR and MMT are lower in OHS patients than in controls. The depression and anxiety scores of OHS patients are higher than in controls. These findings suggest that the management of OHS needs a multidisciplinary approach. It is suggested that short term PAP therapy can have positive effects on neurocognitive functions, depression and anxiety but further multicentre, prospective studies with large number of cases are needed to evaluate the effect of long term PAP therapy on these parameters.

## Abbreviations

ABG, artery blood gas analysis; BDS, beck depression scale; BMI, body mass index; BP, bodily pain; COPD, chronic obstructive pulmonary disease; ECR, enhanced cued recall; ESS, epworth sleepiness scale; GH, seneral health perception; MH, mental health; MMT, mini mental test; MOCA, montreal cognitive assessment scale; OHS, obesity hypoventilation Syndrome; OSAS, obstructive sleep apnea syndrome; PAP, positive airway pressure; PF, physical functioning; PSG, polysomnography; RE, role limitations due to emotional problems; RP, role limitations due to physical problems; SF, social functioning; SF-36, short form-36; STAI 1-2, state-trade anxiety; VT, energy/vitality; WCST, wisconsin card sorting test.
